# Moyamoya disease: A clinical spectrum, literature review and case series from a tertiary care hospital in Pakistan

**DOI:** 10.1186/1471-2377-9-15

**Published:** 2009-04-15

**Authors:** Sana Shoukat, Ahmed Itrat, Ather M Taqui, Moazzam Zaidi, Ayeesha K Kamal

**Affiliations:** 1 Department of Medicine, Aga Khan University, Karachi, Pakistan; 2 Department of Biological and Biomedical Sciences, Aga Khan University, Karachi, Pakistan

## Abstract

**Background:**

Moyamoya is a rare cerebrovascular disease of unknown etiology. The data on moyamoya disease from Pakistan is sparse. We report a case series of 13 patients who presented with moyamoya disease to a tertiary care hospital in Pakistan with a national referral base.

**Methods:**

We conducted a retrospective review of thirteen patients who presented to The Aga Khan University and diagnosed with "Moyamoya Disease" during the period 1988 – 2006. These patients were identified from existing hospital database via ICD-9 codes. A predesigned questionnaire containing information about clinical presentation, management and neuroimaging was administered to all identified patients.

**Results:**

There were seven males and six females. Mean age at presentation was 16.5 years and a female predominance was found in the pediatric age group (n = 10, 71.4%). Stroke (n = 11, 84.2%) was the most common presentation with motor deficit being the universal cortical symptom. Fever was a common symptom in the lower age groups (n = 4, 51.7%). Cerebral Angiography and Magnetic Resonance Angiography showed bilateral involvement of the vessels in eleven patients while unilateral in two. Subarachnoid and interventricular haemorrhage appeared in 2(15.4%) adults. Twelve (92.3%) patients were discharged as independent with minor deficits regardless of therapeutic modality. Only three (23.0%) patients underwent surgery whereas the remaining were managed conservatively.

**Conclusion:**

Physicians when dealing with childhood strokes and characteristic deficits in adult population should consider Moyamoya disease.

## Background

Moyamoya disease is a rare progressive vaso-occlusive disorder of an unknown etiology. It is characterized by progressive stenosis of terminal portions of internal carotid arteries bilaterally, and the main trunks of Anterior and Middle Cerebral Artery, and is associated with collateral vessels at the base of the brain ('moyamoya' vessels).[1] When similar clinical manifestations are associated with an underlying disorder, it is refered to as Moyamoya syndrome. However, since the diagnostic criteria of this disease are mainly based on angiographic findings, it is recommended that the term Moyamoya 'syndrome' should be avoided at best.[2] Previously thought to be prevalent only in Japan, cases have now been reported from across the globe.[3,4] However, majority of the cases are reported in Asia and other non-Caucasian regions. [2].

Moyamoya disease has remained rather unexplored in Pakistan. So far only one case series of four patients has been described, while a few case reports exist.[5-7] Since several studies have reported the occurrence of stroke in young in this region [8], it could be questioned whether Moyamoya disease is under-diagnosed in Pakistan.

The patterns of presentation of Moyamoya disease reported in the literature so far have shown several consistent features, such as hemiparesis, monoparesis or sensory disturbances reflecting TIA in children, or intracerebral, intraventricular and/or sub-arachnoid haemorrhage in adults. Whether these particular patterns of presentation are universal, and if not, what differences exist from the conventional findings are some of the questions that remain to be answered with respect to this particular disease, The aim of this article is to describe our clinical experience with Moyamoya disease in a tertiary care hospital in Pakistan.

## Methods

We report 13 patients presenting to The Aga Khan University diagnosed with "Moyamoya Disease" during the period 1988 – 2006, according to the retrospective search query in the hospital database. Diagnosis was based on characteristic radiological findings on Cerebral Angiogram or MRI/MRA. All patients received an MRI and MRA, and in those whose findings were suggestive but not clear or those that required further intervention received a 4 vessel cerebral angiogram. The data was collected during the month of August 2006. A questionnaire containing fields of demographics, clinical features, imaging, hemodynamic studies, procedures, treatment and follow up was designed and filled for each case. A MeSH search was conducted on the MEDLINE database using key words "*Moyamoya Disease*" [MeSH], "*Moyamoya Disease*" [MeSH] AND "*Pakistan*" [MeSH]. Also, a comprehensive literature search was performed to assess the differences between clinical pictures and outcomes of moyamoya disease in various geographical locations. The local hospital ethical review committee approved this study.

## Results

The hospital database revealed 13 patients (7 males and 6 females) admitted over the past 20 years (1986–2006). Of the 13 patients reported, 7 (53.8%) belonged to the age group 0–12 yrs, 3 were in the range 13–25, while 3 (23.0%) patients were over 26. Mean age at presentation was 16.57 years and the age of presentation was the age of 1^st ^symptom in 11 (84.6%) patients. The youngest age group (0–12 years) comprised only of females.

Table 1 shows the symptomatic checklist and outcomes for each patient. The clinical presentation included fever, stroke, headache and seizures. All the patients with sudden onset of stroke (n = 11, 84.6%) had a motor deficit making it the most common cortical symptom. Blindness and aphasia were present in 1 patient each. Fever was a common presentation especially in the lower age groups, occurring in 4 (30.7%) of the patients. 6 (46.1%) cases presented with seizures, with one having a generalized tonic-clonic. 1 (7.7%) patient developed bilateral hemiparesis and seizures while suffering from encephalitis. None of the patients reported any inherited disorders in the family. 3 (23.0%) patients were found to suffer from repeated TIAs as they presented with intermittent weakness.

**Table 1:** Patterns of presentation

**Patient**	**Age/years**	**Sex**	**Fever**	**Headache**	**Seizure**	**Stroke**	**Hemorrhage**	**Bilateral**	**Repeated TIAs**	Surgery*	**Outcome**
1	6	F	Y	Y	N	Y	N	N	N	N	Dx

2	3.5	M	Y	N	Y	Y	N	Y	N	N	Dx

3	18	M	Y	N	Y	Y	N	N^a^	N	Y	Dx

4	25	M	Y	Y	N	Y	N	Y	N	N	Died

5	12	M	N	N	N	Y	N	Y	N	N	Dx

6	40	M	N	Y	N	N	Y	Y	N	N	Dx

7	9	F	N	N	Y	Y	N	Y	N	Y	Dx

8	4	F	N	N	N	Y	N	Y	N	Y	Dx

9	40	M	N	Y	N	N	Y	Y	N	N	Dx

10	0.5	F	N	N	Y	Y	N	Y	Y	N	Dx

11	10	F	N	Y	Y	Y	N	Y	Y	N	Dx

12	17	M	N	Y	N	Y	N	Y^a^	Y	N	Dx

13	30	F	N	Y	Y	Y	N	Y	N	N	Dx

Y: yes; N: No; F: Female; M: Male; Dx: Discharged

*Revascularization surgery

^a ^based on symptoms

Past medical history of 1 (7.7%) of the patients revealed encephalitis following post natal sepsis, while 2 (15.4%) cases had past neurological history, with one having cognitive deficits and the other being aphasic since birth. 2 (15.4%) of the patients had a history of cardiac defects at birth, which resolved spontaneously in both the cases. A 40-year-old male who presented with occipital headache had a history of repeated headaches, for which he was taking analgesics off and on. 1 (7.7%) patient had a post-partum state, having delivered two weeks back. She had a history of repeated abortions.

**Figure 1:**
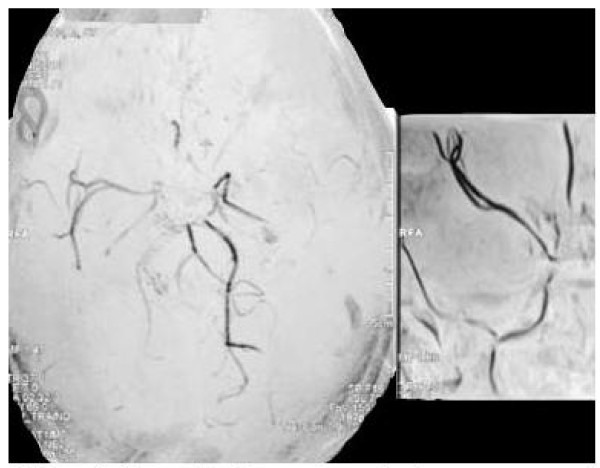
**Magnetic Resonance Angiogram showing bilateral stenosis suggestive of Moya Moya Disease**.

**Figure 2:**
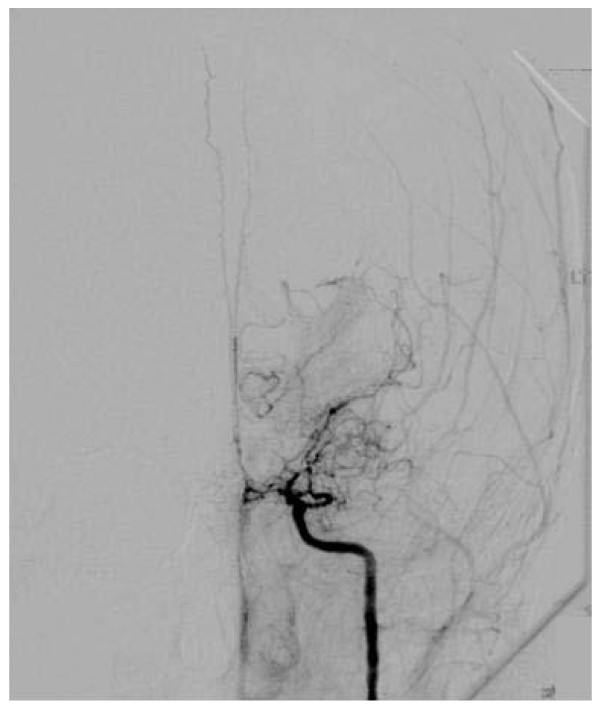
**Angiogram showing stenosis of the supraclinoid ICA and MCA consistent with Moya Moya Disease**.

**Figure 3:**
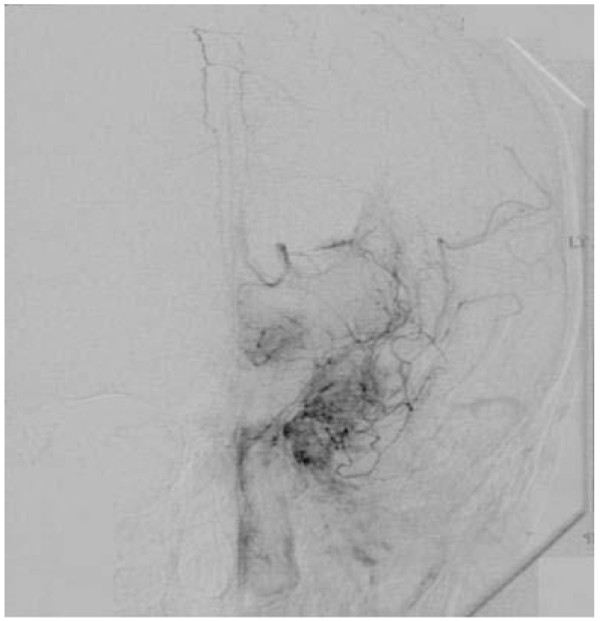
**Cerebral Angiogram showing the "puff of smoke" that comprises the abnormal collaterals**.

MRI and MRA was performed in all cases 7 (53.9%) patients underwent Cerebral Angiography either at the time of presentation. Of the 13 patients reported, 11(84.6%) had bilateral involvement of the vessels showing stenosis to complete occlusion on MRA or cerebral angiograms.(Figure 1) The various sites of occlusion and stenosis included the supraclinoid portions of ICA, MCA and ACA at specific segments or at their origins at circle of willis.(Figure 2) 2 (15.4%) cases showed unilateral involvement hence falling under the category of '*probable moyamoya disease'*. Cranial CT and MRI showed subarachnoid and interventricular haemorrhage in 2 adults. Of the remaining 11(84.6%) patients, 4 (30.8%) patients had multiple corticomedullary infarcts while the rest had a co-existing sub-cortical infarct involving the territory of the stenosed/occluded vessel-mostly the ACA or/and the MCA. The classical 'smokey' appearance of moyamoya vessels was seen only in 4 (30.8%) patients. (Figure 3)

EEG was performed in 6 (46.1%) patients. 4 (30.8%) showed diffuse cerebral dysfunction whereas two (15.4%) had an epileptiform activity; with one being focal and the other non-focal.

Lab investigations, including coagulation profiles were performed in all cases. Of the thirteen patients in this study, those that presented with fever and seizures (6;46.2%) got a lumbar puncture. 4 (66.7%) had a normal CSF, 2 (33.3%) had a moderate rise in proteins showing a level of 70 mg/dl. None had a positive culture.

Ancillary lab results, for most parts, were unremarkable. None of the patients showed a hypercoagulable state, and ANA, ASMA, Anti-DSDNA, and Anticardiolipin antibodies were negative in all patients. 1 (7.7%) patient had mildly elevated Serum Homocysteine levels (12.74, patient D).

3 patients underwent surgical procedures. A 9 yr old had an EC/IC bypass and two patients, aged 18 and 4 underwent EDAS. 1 (7.7%) patient developed an extradural hematoma right after the procedure which was removed promptly and the patient improved. All patients were discharged once stable after the procedures.

Of the 10 patients who underwent conservative management. 2 (15.4%) received steroids at the discretion of the treating physician. Anticonvulsants were given to the patients who had presented primarily with a seizure or developed them during hospital stay. Drug levels were monitored for these patients. Febrile patients were given antiviral and systemic antibiotics until CSF culture and Herpes PCR results were received. IV Mannitol was reserved for patients with deteriorating neurological status or increased intracranial pressure in the setting of haemorrhage.

12 (92.3%) patients were eventually discharged with improved GCS and minor neurological deficits. These included limb weakness, partial aphasia and headaches. 1 (7.7%) adult male had a sudden deterioration of symptoms after bilateral stroke, who expired after a brief coma.

## Discussion

For several decades since its discovery by Takeuchi and Shimizu in 1957 [9], Moyamoya disease was considered to be mainly confined to certain ethnic groups, particularly the Japanese. As more cases were discovered worldwide, interest into understanding its etiology, pathogenesis and clinical course also became more profound. So far, approximately 6000 cases of Moyamoya disease have been reported across the globe, with almost two-thirds confined to Japan. [9,10] However, a number of cases from other countries including Korea, China, India and even the United States have now been added to the literature.[11-14]

Moyamoya disease is a cerebrovascular pathology, characterized by unilateral or bilateral stenosis or occlusion of intracranial segment of internal carotid artery (ICA) and the proximal parts of anterior and middle cerebral arteries. Subsequently, parenchymal, leptomeningeal and transdural collaterals develop in the ischemic brain as a response to hypoxia.[15-17] Literally meaning "a puff of smoke", the name moyamoya disease was coined on the basis of radiological finding by Suzuki and Takaku [1], when the abnormal collaterals appeared to give a hazy, 'cigarette smoke' appearance on a cerebral angiogram.

The pathological findings as seen on autopsy cases, irrespective of age, suggest involvement of distal portion of carotid arteries and the proximal portions of the anterior and middle cerebral arteries. There is extensive intimal thickening with tortuous and duplicated internal elastic lamina of the arteries, and there may also be lipid deposition in the intima. However, an important feature is the lack of inflammatory signs and atheromatous plaque.[9,18]

Etiology of Moyamoya disease is controversial. Even though several linkage studies have shown promising relations with gene loci [19,20], no specific locus has yet been identified. However, these linkage studies, along with the familial occurrence of Moyamoya, point towards a probable genetic basis underlying its etiology. Investigations into understanding the pathogenesis of Moyamoya disease have shown involvement of the CSF basic Fibroblast growth factor (b-FGF) with receptor up-regulation, and TGF beta 1 in altering the cerebral vasculature.[9,21,22] Intimal thickening has also been postulated resulting from altered permeability owing to enhanced prostaglandin release from the arterial smooth muscle.[23]

Our series of thirteen Moyamoya cases is the largest by far in the country. This small number for a 20 year record at one of the largest tertiary care hospital in Pakistan suggests that either the prevalence of Moyamoya disease is low in the country, or that the disorder is seldom brought to clinical attention. The latter could be supported on the grounds that Pakistan, like its neighboring regions, is burdened with the wrath of communicable diseases, which are responsible for the major share of infant and childhood mortality, and hence obscure the actual incidence of non-communicable diseases. Also, it is possible that a clinical variant of Moyamoya disease occurs in this region, which goes by undiagnosed at large.

Our study sample had an almost equal sex distribution, in contrast to the worldwide literature, which suggests a slight female preponderance. Due to scanty local data, the higher occurrence in males in our sample cannot be commented upon conclusively. However, pediatric studies have proven that the overall risk factor for stroke is greater in boys compared to girls.[24]

Moyamoya disease is reported to primarily present at an early age, typically less than 10 years, and is more common in females (M:F 1:1.8). However, a second peak in the fourth decade has also been described in the literature.[25] Our patients showed a consistent finding, with 7 (54%) of the cases under the age of twelve with male adults being predominantly affected.

Conditions such as sickle cell anemia, Neurofibromatosis-1, Down's syndrome, congenital heart defects, Antiphospholipid syndrome, Renal artery stenosis and thyroiditis have been found to be associated with Moyamoya disease in the literature.[26] Our two cases with congenital heart defects support the view of Lutterman et al., who reports five patients with congenital heart defects who developed Moyamoya disease in childhood.[26] It appears from our review that most of our patients had Moyamoya disease rather than the syndrome as none had the known associations except for the congenital cardiac defects mentioned above.

One of our cases had history of cognitive difficulty in school prior to the presenting episode of stroke. Such an intellectual decline has been described but usually after Moyamoya disease is diagnosed.[27] This finding could point to the fact that in addition to the consequences like stroke and seizures, this progressive stenotic process even directly affects intellect in patients.

The clinical features of patients with Moyamoya disease reflect the anatomic territory of the brain affected by the diseased vessel. Yamaguchi et al. in the Annual Report for the special working group of Welfare Ministry for Moyamoya in 1979 described four major types of Moyamoya disease according to clinical manifestations; the hemorrhagic type, the infarction type, TIA type, and epileptic type; with the first two types being the most common. [28]

In our cases, stroke symptoms outnumbered other presenting complaints. All the females presented with stroke as the major complaints, while two males who did not have any symptomatic stroke were found to have intraventricular haemorrhage on CT Scans. This is an inconsistent finding with other Asian studies, which have revealed greater prevalence of hemorrhagic presentation in females [29-33]. Apart from stroke, headache was another consistent feature in our series. The pathophysiological mechanism of headaches in patients with Moyamoya disease remains unclear, but it is presumed to be closely related to cerebral hypoperfusion because it has been reported that headaches disappeared after surgery.[34]

An interesting finding in our cases was a post-infectious presentation of the patients. Hyperthermia induced vasoconstriction has been described to affect luminal diameter of the carotid artery leading to hypoperfusion.[35] Hyperthermia could have led to compromise of blood flow and compromise of the functioning collaterals and trigger the symptoms in four of our cases. No literature exists at present that has probed the association between raised body temperature and occurrence of Moyamoya, and hence the hypothesis so far could only be based on the aforementioned discussion unless conclusive, controlled experiments are carried out.

Despite the reported risks associated with conventional angiography [36], it is still considered the gold standard for diagnosing Moyamoya disease. Magnetic resonance imaging was the most common technique used in our cases. MRA proved to be a helpful diagnostic tool identifying sites of stenosis and demonstrating the collateral vessels at the base of the brain. As a non-invasive procedure, it has been described in the literature as a promising alternative to classical angiography for this arterial disease. [37] Combined with MRI, it gives information about brain parenchyma and hence provides a better correlation of the symptoms with the radiological findings. However, it overestimates occlusion and underestimates presence of collaterals and can lead to higher staging of patients and lead to surgical interventions.[1] Cerebral Angiography remains the gold standard for these patients.

Irrespective of the radiological method used for diagnosis, conditions like von Recklinghausen's disease, Down's syndrome, autoimmune vasculitis, head trauma, meningitis and brain tumors may have a similar angiographic picture and hence can possibly confuse the diagnosis. Also, postpartum cerebral angiopathy and inflammatory angiopathy are two important differential diagnoses that may present with cerebral ischemia and imaging consistent with intracranial vasculopathy mimicking Moyamoya disease.[38,39]

Yamashiro describes three important patterns of EEG in Moyamoya patients- a slow wave pattern, localized slow waves and temporary but prominent "build ups" that are high voltage delta bursts secondary to hyperventilation.[40] However, we did not find these specific patterns in our patients. They either had theta or delta slowing or evidence of epileptiform activity secondary to focal ischemia. This lack of characteristic EEG findings in our patients could contribute to the disagreement with Yamashiro's hypothesis of EEG as a screening tool for this disease. However, it should be remembered that our case patterns are not completely comparable to those in Japan because of supposed differences in hereditary patterns.

Revascularization procedures are currently performed to increase the perfusion to the hypoxic brain tissue. The literature supports these procedures and long-term favorable outcome has been reported in terms of improvement in symptoms and positive angiographic follow-ups in all age groups.[12,41,42] Four types of surgical procedures have been described-indirect procedures including encephalo-duro-arterio-myo-synangiosis (EDAMS), direct revascularization via the superficial temporal artery and the middle cerebral artery (STA-MCA) bypass, combined approaches and rarely, denervation of cerebral vasculature. In general, pediatric cases benefit from indirect revascularization procedures and the direct bypass is useful in most of adult cases.[43] Our series demonstrates a successful direct procedure on a child and EDAMS on a child and an adult case. More patients could have benefited from revascularization procedures to alleviate the symptoms but cost and loss to follow-up were major barriers in our setting. It is difficult to comment on specific rationales for strategy in each case since the setting is a fee for service tertiary care centre. These decisions were made not purely on a scientific basis, individual affordability, practicing surgeons comfort level and expertise with procedure all played into the treatment decisions. In general, intervention for moya moya is offered as the last resort and is often an indirect vascularization. Expertise for direct vascularization is not available in Pakistan – these procedures are performed with visiting neurosurgeons from other countries, if at all.

Surgery results in decreased incidence of TIAs and major complete strokes in the patients. However, severe stroke preoperatively proves to be a poor prognostic factor for the outcomes of the procedure.[44] Potential risks include complications in handling the thin cortical vessels, postoperative haemorrhage from the neovascular channels and a delay or even failure of effective resupply of blood.[20,45]

Prognosis of patients with Moyamoya disease is found to be related to age and the type of presentation. Kim et al have demonstrated less favorable outcome in younger age groups (< 3 yrs) with severe presentations and increased incidence of preoperative strokes.[46] However, hemodynamic improvement after surgical procedures appears to be similar in all age groups. TIA and epileptiform clinical pictures have a better long-term outcome when compared to infarctions.[20] In pediatric patients, the TIA groups have shown better outcomes in terms of school performance, higher IQs and lesser incidence of severe disabilities. The immediate outcome for our series was reasonably good. All patients save one had total or near total recovery, and those who underwent surgery also had positive post operative follow ups. However, due to the lack of longitudinal data, the functional outcomes of all these patients cannot be commented upon with certainty.

Moyamoya disease, although a rare disorder in the Pakistani population, is probably underdiagnosed. Diagnosis requires expensive radiological studies and trained neurophysicians that are not available in most parts of the country. Additionally, the lack of neurosurgeons and their expertise in highly sophisticated procedures involved in the treatment makes it further difficult to manage patients.

## Conclusion

Stroke symptoms were frequently noticed in Moyamoya patients, most of the presenting features were compatible with reports from other geographic regions with few exceptions. Only 13 patients presented in 20 years at a tertiary care hospital in Pakistan, which indicates that either the prevalence of Moyamoya is very low in this region, or the disorder is seldom brought to medical attention. This emphasizes the need to consider Moyamoya disease in young patients and children presenting with stroke symptoms.

## Competing interests

The authors declare that they have no competing interests.

## Authors' contributions

SS and AI conceptualized the study and were involved in the study design. AT and MZ collected the data. AI and SS were involved in the data analysis and data interpretation. SS, MZ, AI and AK prepared the manuscript. AK provided critical feedback and guidance and was responsible for the study's ongoing management. All authors read and approved the final manuscript.

## Pre-publication history

The pre-publication history for this paper can be accessed here:


